# Abdominal angina due to recurrence of cancer of the papilla of Vater: a case report

**DOI:** 10.1186/1752-1947-3-9314

**Published:** 2009-12-02

**Authors:** Marco Biolato, Maria Letizia Gabrieli, Antonello Parente, Simona Racco, Melania Costantini, Lorenzo Bonomo, Gian Ludovico Rapaccini, Giovanni Gasbarrini, Antonio Grieco

**Affiliations:** 1 Department of Internal Medicine, Catholic University of Rome, 8 Largo A Gemelli, 00168 Rome, Italy; 2 Department of Radiology, Institute of Internal Medicine, Catholic University of Rome, 8 Largo A Gemelli, 00168 Rome, Italy

## Abstract

**Introduction:**

Abdominal angina is usually caused by atherosclerotic disease, and other causes are considered uncommon. This is the first report of a case of abdominal angina secondary to neoplastic vascular stenosis caused by local recurrence of an adenocarcinoma of the papilla of Vater.

**Case presentation:**

An 80-year-old woman of Caucasian origin presented with abdominal pain and diarrhea. She had undergone a pancreaticoduodenectomy for adenocarcinoma of the papilla of Vater four years earlier. Computed tomography revealed a mass surrounding her celiac trunk and superior mesenteric artery. Her abdominal pain responded poorly to analgesic drugs, but disappeared when oral feedings were withheld. A duplex ultrasonography of the patient's splanchnic vessels was consistent with vascular stenosis. Parenteral nutrition was started and the patient remained pain free until her death.

**Conclusion:**

Pain relief is an important therapeutic target in patients with cancer. In this case, abdominal pain was successfully managed only after the ischemic cause had been identified. The conventional analgesic therapy algorithm based on nonsteroidal anti-inflammatory drugs and opioids had been costly and pointless, whereas the simple withdrawal of oral feeding spared the patient of the discomfort of additional invasive procedures and allowed her to spend her remaining days in a completely pain-free state.

## Introduction

Chronic mesenteric ischemia is an under-recognized cause of postprandial abdominal pain. In over 90% of all cases, this abdominal angina is caused by atherosclerotic occlusion or severe stenosis of mesenteric arteries [1,2]. The diagnosis is usually based on the results of imaging studies, such as duplex ultrasound, traditional angiography, magnetic resonance angiography, and computed tomography (CT) angiography [3,4].

There are rare cases where mesenteric ischemia is unrelated to atherosclerotic stenosis. This report describes a very unusual cause of abdominal angina secondary to non-atherosclerotic mesenteric stenosis. The correct diagnosis of the cause of stenosis allowed the attending physicians to provide individualized therapy that had a positive impact on the patient's quality of life.

## Case presentation

An 80-year-old italian woman of Caucasian origin presented to the emergency room at the Catholic University of Rome with severe abdominal pain and bloody diarrhea. Her symptoms, which had developed over the last four months, consisted of unrelenting lower abdominal pain that began 30 minutes after eating and lasted for about three hours. It was unrelieved by bowel movements or changes in position. For this reason, the patient reduced her food intake, and her weight decreased by 5 kg. The day before admission, bloody diarrhea developed.

The patient had a history of arterial hypertension, hiatal hernia, bilateral hearing loss due to chronic otomastoiditis, and polyarthritis (cervical, dorsal, and lumbosacral spondylosis; bilateral osteoarthritis of the hip). She had undergone open surgical cholecystectomy for gallstones in 1959. In 1998, she was hospitalized for rectal bleeding caused by acute diverticulitis. In 2004, she was diagnosed with adenocarcinoma of the papilla of Vater and had a cephalic pancreaticoduodenectomy. The pathological examination revealed a moderately differentiated intestinal-type adenocarcinoma measuring 1.5 cm in diameter that had invaded the muscle layers of the duodenal wall. The margins were tumor-free, and two lymph nodes were negative for malignancy. During that hospitalization, she developed paroxysmal atrial fibrillation that was converted to a normal sinus rhythm with amiodarone. Her medications included zofenopril (7.5 mg/day), manidipine (10 mg/day), esomeprazole 20 mg/day, celecoxib (400-600 mg/day), and acetaminophen plus codeine (500 mg plus 30 mg/day). She had no known drug allergies.

During admission, she was alert and oriented with normal vital signs (blood pressure, 150/70 mm Hg; heart rate, 80 beats per minute; temperature, 36.8°C). The lower abdomen was tender, but there were no signs of peritonitis. Although the bowel sounds were decreased, passage of flatus and feces was normal. There was no palpable organomegaly. The patient's lungs were clear on auscultation and a systolic ejection murmur (2/6) was heard over her aortic area. Admission laboratory tests revealed: hemoglobin 12.1 g/dl; white-cell count 15,290/mm^3 ^(neutrophils 86%); platelet count 317,000/mm^3^; total protein 5.6 g/dl; albumin 3.2 g/dl. Serum electrolytes, creatinine, glucose, bilirubin, alanine aminotransferase, gamma glutamyl transferase, and amylase levels were within normal limits, as were the prothrombin and partial thromboplastin time. Plain films of the abdomen revealed no free intraperitoneal air or air-fluid levels, and the chest x-ray excluded the presence of pneumonia or nodules. Immediately after admission she was placed on an NPO (*nil per os*) regimen with total parenteral nutrition, and within eight hours her symptoms completely disappeared.

**Figure 1:**
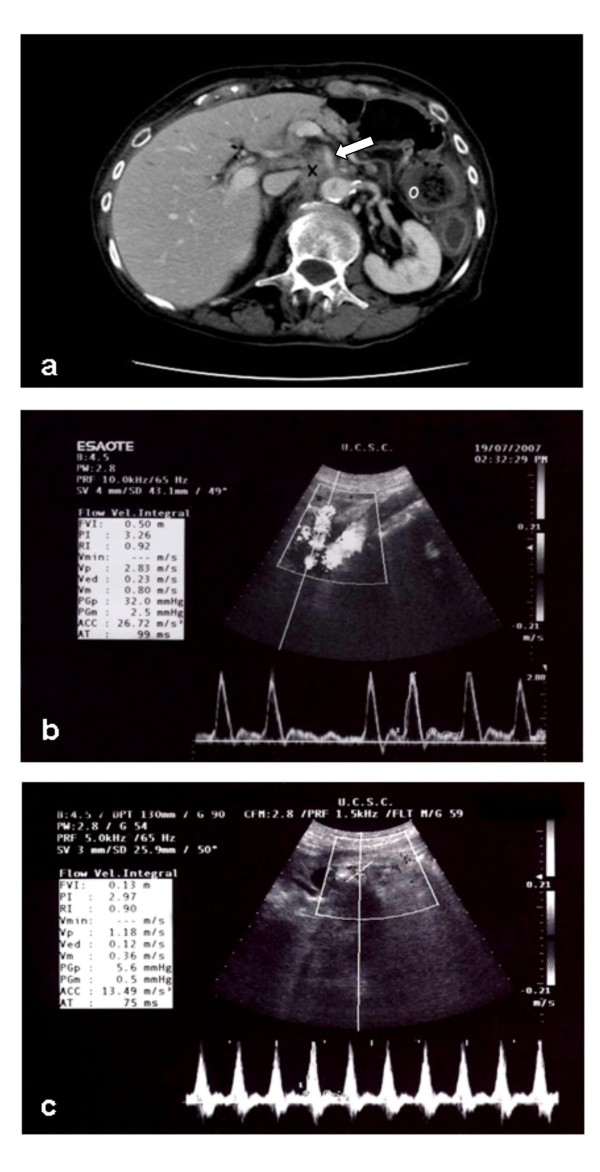
**(A) Contrast-enhanced abdominal CT scan**. Axial image of the origin of the superior mesenteric artery (↑) from the abdominal aorta. A hypodense mass (X) envelops the origin of the artery. Bowel loop thickening (O) is also evident. **(B) **Duplex ultrasound assessment of flow through the superior mesenteric artery. **(C) **Duplex ultrasound assessment of flow through the celiac trunk.

Abdominal CT (Figure 1A) revealed a hypodense mass measuring 4 × 4 cm in the space between the vena cava and the aorta, at the level of the origin of the celiac trunk. The mass extended caudally for about 4 cm, enveloping the origin of the superior mesenteric artery, the left renal vein at its confluence into the inferior vena cava, and the origin of the right renal artery, and anteriorly to the confluence of the splenic and mesenteric veins. Enlarged lymph nodes (1 cm) were observed at the hepatic hilum and in the intercavoaortic and left para-aortic spaces. Thickened bowel loops were also seen. This picture was consistent with local recurrence of the neoplastic disease.

A week later, oral feedings were resumed and the patient once again experienced diffuse abdominal pain and diarrhea. The pain responded poorly to conventional analgesics (scopolamine 20 mg IV, acetaminophen 500 mg PO, tramadol 37.5 mg PO t.i.d., fentanyl 25 μg transdermally), but it disappeared promptly when oral feedings were withdrawn.

A duplex ultrasound examination was performed to assess the mesenteric circulation (Figure 1B and Figure 1C). To the extent that it could be explored, the artery showed no evident stenoses on B-mode or color- and power-Doppler evaluation. The superior mesenteric artery was characterized by a high peak systolic velocity (283 cm/sec) that was consistent with vascular stenosis. Blood flow in the celiac artery was turbulent, and the peak systolic velocity was also high (118 cm/sec). The patient was started on subcutaneous enoxaparin (4000 U/day) and transdermal nitroglycerin (10 mg/day, from 08.00 to 20.00). Since oral feedings could not be resumed, a central venous catheter was inserted for prolonged parenteral nutrition.

In view of the tumor histotype and the age and general condition of the patient, there was no indication for systemic chemotherapy and the patient was transferred to a hospice. She remained pain-free without any form of analgesics and had a relatively good quality of life until her death three months later.

## Discussion

This is the first report of abdominal angina secondary to neoplastic vascular stenosis caused by local recurrence of an adenocarcinoma of the papilla of Vater. However, there have been reports of chronic mesenteric ischemia that developed shortly after cephalic pancreaticoduodenectomy, which involves resection of the gastroduodenal artery. In the presence of atherosclerotic stenosis of the celiac artery, the procedure can lead to ischemia of the liver, pancreas, and biliary tree [5-8]. For this reason, when pancreaticoduodenectomy is being planned for an elderly patient with known risk factors for atherosclerosis or with manifestations of atherosclerotic disease in other districts, the splanchnic circulation must be subjected to a thorough preoperative assessment based on conventional CT or CT angiography. Magnetic resonance angiography is also emerging as a useful diagnostic tool in this setting [9]. If stenosis of the celiac-mesenteric axis is found (even in the absence of symptoms), preoperative stenting is advisable [6,7]. In rare cases, younger patients may present evidence of stenosis caused by an anomalously inserted arcuate ligament, which compresses the celiac trunk [10].

In view of the age of the patient as well as her poor prognosis, our main priority was the quality of her remaining life. Additional invasive investigations (conventional angiography or magnetic resonance angiography) were deferred, as was percutaneous angioplasty with stent placement, which is considered a low-risk procedure but is nonetheless invasive.

Duplex ultrasound assessment of the mesenteric arteries is a valuable diagnostic tool that has gained widespread acceptance over the past two decades. Its noninvasiveness and portability are distinct advantages for patients who are seriously ill [11].

Stenotic and occlusive lesions are manifested by turbulence and high flow velocities in the proximal portion of these arteries. Peak systolic velocity and end-diastolic velocity have been validated against angiographic findings, and they have proved to be highly accurate indicators of significant (≥50%) stenosis of the proximal superior mesenteric artery or celiac trunk stenosis (overall accuracies >90% and >80%, respectively). For the superior mesenteric artery, a peak systolic velocity ≥ 275 cm/sec, an end-diastolic velocity ≥45 cm/sec or no flow signal are the most used duplex velocity criteria for vascular stenosis, while for the celiac trunk, a peak systolic velocity ≥200 cm/sec, an end-diastolic velocity ≥55 cm/sec or no flow signal are used [1].

In our case, the imaging studies revealed that the cause of the stenosis was not atherosclerosis but rather a local recurrence of a malignant tumor. This information allowed us to focus our attention on the need of the patient to effectively control pain. Surgical revascularization of the bowel and percutaneous angioplasty with stenting are the most effective approaches for treating chronic mesenteric ischemia [12], but neither was deemed feasible in our patient. There is no evidence supporting the use of conservative medical treatment of chronic mesenteric ischemia. Suggested treatments include eating small meals, proton pump inhibitors (to decrease the oxygen demands of the gastric mucosa), refraining from smoking, and vasodilator drugs (to decrease vasospasm) [2]. Our patient presented with severe postprandial abdominal pain, which could not be controlled with scopolamine, acetaminophen, tramadol, or even fentanyl, and withdrawal of oral feeding was therefore our only option. Adequate nutrition was supplied parenterally, and the patient was given enoxaparin and nitroglycerin. Anticoagulant therapy is widely employed in acute mesenteric ischemia and is even considered a possible alternative to traditional surgical bypass, embolectomy, and percutaneous angioplasty with vascular stenting [13]. Sonographic studies have shown that acute administration of nitrates is followed by significant dilatation of the superior mesenteric artery and hepatic artery [14].

## Conclusion

Pain relief is an important therapeutic target among patients with cancer. In this case, abdominal pain was successfully managed only after the true cause of the patient's ischemia had been identified. The conventional analgesic therapy algorithm based on nonsteroidal anti-inflammatory drugs and opioids would have been costly and in this case pointless, whereas the simple withdrawal of oral feedings spared the patient the discomfort of additional invasive procedures and allowed her to spend her remaining days in a completely pain-free state.

## Consent

Written informed consent was impossible to obtain because the patient had died and no living relative could be found. However, we have ensured that all reasonable attempts to gain consent were made and that the patient is anonymous.

## Competing interests

The authors declare that they have no competing interests.

## Authors' contributions

MB, MLG, SR and AG clinically managed the patient and were a major contributor in writing the manuscript. AP and GLR performed the duplex evaluation and interpreted the data according to diagnostic standard for abdominal angina. MC and LB performed the CT scan and diagnosed the neoplastic local recurrence. GG made an important contribution to interpreting the clinical picture. All authors read and approved the final manuscript.
